# Substantial Improvement in a Patient with Subacute Sclerosing Panencephalitis: An Unusual Case Report

**DOI:** 10.5334/tohm.972

**Published:** 2024-11-21

**Authors:** Sneh Jain, Shweta Pandey, Ravindra Kumar Garg, Swansu Suresh Kumar Batra

**Affiliations:** 1 Department of Neurology, King George’s Medical University, Lucknow, India

**Keywords:** SSPE, Interferon, Improvement

## Abstract

**Background:**

Subacute Sclerosing Panencephalitis (SSPE) is a fatal disorder marked by gradual cognitive and motor deterioration, leading to death typically within 1–3 years.

**Case report:**

A 20-year-old woman with progressive abnormal behaviour, forgetfulness, and involuntary movements showed significant improvement after treatment with interferon and isoprinosine. Initially severely cognitively impaired and dependent, she regained independence and demonstrated marked cognitive enhancement, her MMSE improved from 15 to 28 and reduced myoclonus. Her progress was sustained over three years, substantially enhancing her quality of life.

**Discussion:**

This SSPE case shows significant improvement in disability. Early identification of such cases is crucial for improved prognostic counselling for families.

## Background

Subacute Sclerosing Panencephalitis (SSPE) represents a rare, progressive neurodegenerative disorder typically resulting in fatality. It stems from a persistent infection with a mutant strain of the measles virus. The disease is characterized by a slow yet inevitable decline in cognitive and motor functions, culminating in death within one to three years from its onset [1]. Notwithstanding, there are exceptional instances where patients have exhibited spontaneous remission or a positive response to therapeutic interventions.

## Case Report

A 20-year-old married woman with an education up to the tenth grade presented with a one-year history of abnormal behaviour and forgetfulness. She also exhibited abnormal jerky, involuntary movements of the left upper limb for the past ten months. The abnormal behaviour included aggression, socially inappropriate actions, disinhibition, misplacing belongings, and difficulty performing daily household tasks. She would make frequent errors in preparing food and day-to-day chores. Subsequently, she began experiencing transient jerky movements of her left-hand fingers, resulting in the dropping of objects, which progressed to involve the entire left side of her body, leading to frequent falls and the eventual need for assistance while walking. Because of her behaviour and frequent falls, she was left by her husband. Her father started taking care of her afterward. She had a normal birth and developmental history, although she was not vaccinated for measles and had no history of measles during childhood.

On examination, her general physical condition appeared normal, and her Mini-Mental Status Examination (MMSE) score was 15 out of 30, indicating cognitive impairment. Detailed mental function testing showed severe deficits in attention, concentration, memory, and visuospatial integration, with borderline deficits in language function, planning, and execution. She had unilateral myoclonic jerks on the left half of her body, involving her neck, trunk, left arm, and leg, leading to falls. (Video 1) No abnormalities were noted in cranial nerve and motor examinations, and no Kayser-Fleischer ring was observed.

**Video 1:** Segment 1: Upon admission, video reveals frequent myoclonic jerks affecting the left side of the body (neck, trunk, arm, and leg), leading to recurrent falls and requiring support for ambulation. Segment 2 and 3: At the ten-month follow-up, the patient exhibits subtle myoclonic jerks in the left arm while seated, and demonstrates significant improvement in mobility with independent walking and no falls. Segment 4: Ten months post-treatment, the patient is seen dancing enthusiastically, although reduced movement on the left side of the body is still noticeable.

**Figure 1:**
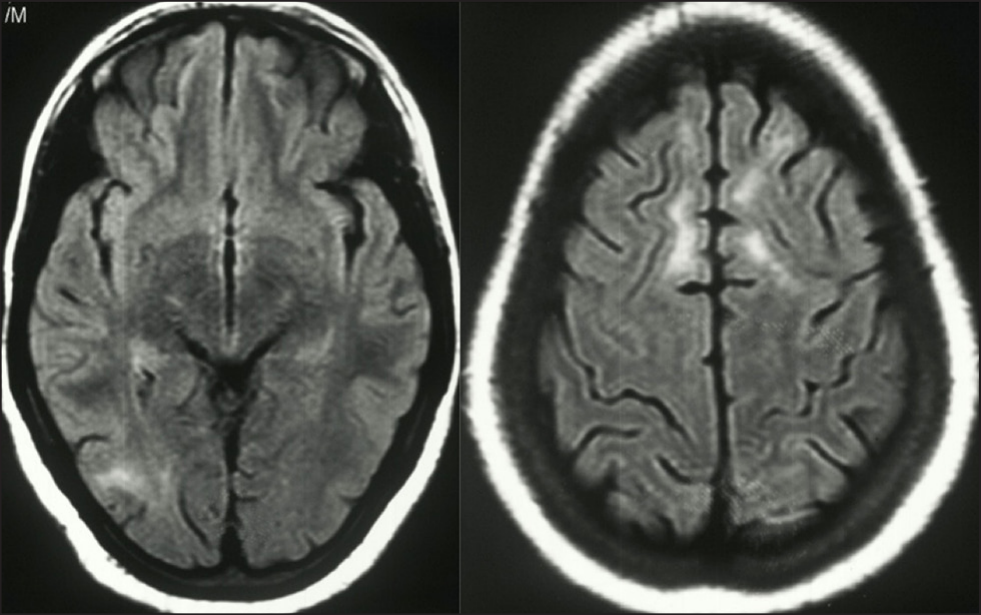
The brain MRI reveals white matter hyperintensities on T2 FLAIR imaging, primarily affecting the right occipital region, bilateral high frontal regions, and additional areas in the left frontal region.

**Figure 2:**
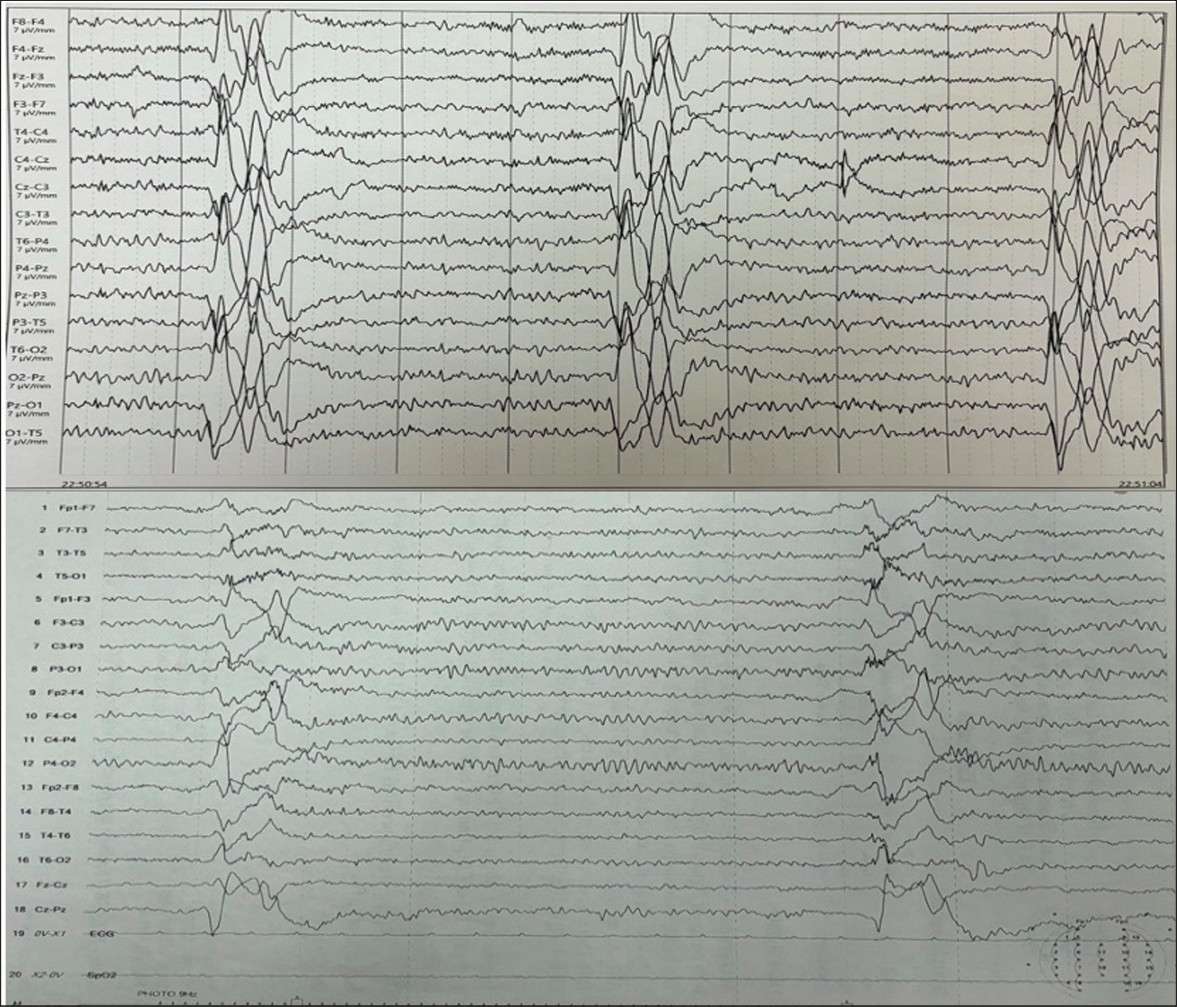
**(Upper panel)** The baseline EEG demonstrates abnormal activity, characterized by periodic generalized polyspike and wave discharges of moderate amplitude (350–450 microV), recurring at regular 3-second intervals. **(Lower panel)** Follow-up EEG: Improved polyspike and wave discharges (100–150 microV) now occurring every 5 seconds, indicating reduced abnormal activity.

Brain MRI revealed signal alterations in the bilateral frontal lobes and the right occipital region. (Figure 1) All blood tests were normal, including complete blood count, renal and liver function, plasma ammonia, thyroid function, anti-thyroid peroxidase antibodies, and serum vitamin B12 levels. Cerebrospinal fluid analysis showed a mildly elevated cell count, normal glucose, and protein levels, with elevated IgG measles antibodies in the CSF. CSF autoimmune panel and screening tests for neurosyphilis were negative. The electroencephalogram displayed periodic generalized poly-spike and wave discharges with a frequency and amplitude suggestive of an encephalopathic process. (Figure 2: Upper panel)

Treatment commenced with interferon alpha-2b, administered intrathecally as six million units weekly for the first six weeks, followed by three million units monthly for six months. Additionally,1500 mg of isoprinosine was prescribed orally in divided doses. No other immunomodulator drugs have been given. After 12 months of treatment, the patient demonstrated notable cognitive improvement, with her MMSE score improving to 28 out of 30. She could make food, start making decisions for her family, communicate with her family members, and dance. Myoclonus ameliorated markedly, and she experienced no further falls. (Video 1) She started living with her husband again after significant improvement in her symptoms. Follow-up EEG showed a decrease in the frequency and amplitude of the periodic complexes. (Figure 2: Lower panel) After three years of follow-up, the patient has not deteriorated again.

## Discussion

SSPE typically follows a progressive and fatal course. However, remission in the course and prolonged survival have frequently been reported. Our case was unique as there was a very significant reversal and achieved much improved mental abilities and motor function.

Several studies in the past have indicated that while SSPE’s course is highly variable, proactive and intensive treatment with antivirals and immunomodulators may contribute to extended survival and periods of remission. A comprehensive literature review on the clinical presentation, treatment, and outcomes of SSPE cases reveals long survival or substantial Improvement with different treatment approaches. (Table 1) [2,3,4,5,6,7,8,9,10,11,12,13,14,15].

**Table 1:** Clinical, Imaging, Treatment, and Follow-Up Characteristics of Published SSPE Cases* Demonstrating Long Survival or Substantial Improvement.**


REFERENCE	COUNTRY	AGE/SEX	MEASLES VACCINATION	CHILDHOOD MEASLES	DURATION OF ILLNESS BEFORE PRESENTATION	CLINICAL PRESENTATION	DISABILITY AT PRESENTATION	NEURO-IMAGING	BRAIN BIOPSY	TREATMENT GIVEN	COURSE OF ILLNESS	OUTCOME

Valente et al. 2021 [2]	Italy	17/M	NA	Measles infection at 2 months	4 months	Seizures Progressive encephalopathy Retinitis Periodic myoclonus	She needed help while walking and changing posture	Periventricular parieto-occipital T2/FLAIR hyperintensity	NA	Antiepileptics Methisoprinol and ribavirin Later Ketogenic diet	After 1 year, she improved	She became independent Able to read and write

Sonoda et al. 2021 [3]	Japan	10/M	NA	13 months of age	2 months	Myoclonus	NA	Periventricular T2/FLAIR hyperintensity Progressive cerebral atrophy	NA	Intraventricular interferon-α and ribavirin	14 years of follow-up	Patient remained stable through follow up

Nathan et al. 2019 [4]	India	8/M	Not vaccinated	At 2 years of age	NA	Myoclonus Repeated falls	Akinetic mute stage	Periventricular T2/FLAIR hyperintensity	NA	Antiepileptics Isoprinosine, ribavirin, and lamivudine Later Ketogenic diet	11 months	Myoclonus stopped Started speaking EEG normalised

Kwak et al. 2019 [5]	South Korea	13/M	Single dose of MMR vaccine	At 2 years of age	NA	Myoclonus Progressive encephalopathy	Akinetic mute stage	Normal	NA	Intraventricular interferon-α and inosiplex	13 years	Bedridden but able to speak

Eroglu et al. 2008 [6]	Turkey	Out of 19 patients, 10 had survival beyond three years. These patients, mostly males aged 14 to 22, survived between 36 and 160 months. Treatments included isoprinosine, often with α-interferon, but all eventually experienced disease progression and succumbed.										

Prashanth et al. 2006 [7]	India	SSPE generally has a progressive, fatal course, yet a minority of patients exhibit long-term stabilization or remission, surviving over 3 years. This study of 19 such cases reveals variable presentations, prolonged stabilization/remission phases, and occasional functional recovery,										

Miyazaki et al. 2005 [8]	Japan	8/M	NA	NA	Sudden	Myoclonus Progressive encephalopathy	NA	Progressive brain atrophy	NA	Intrathecal interferon-α	13 years	Akinetic mute stage Initially greatly improved quality of life for 7–8 years

Kurata et al. 2004 [9]	Japan	17/F	NA	NA	NA	Myoclonus Progressive encephalopathy	Walking difficulty	NA	NA	Intrathecal interferon-α and inosiplex	6 months	Patient improved and was able to speak and walk

Risk and Risk 2003 [10]	USA	6/F	NA	NA	NA	Repeated drop attacks Mental decline	Walking difficulty	Periventricular T2/FLAIR hyperintensity	NA	Antiepileptics only	3 months	Seizures controlled Able to walk Started attending school

Yazaki et al. 2000 [11]	Japan	24/F	Not vaccinated	NA	8 years	Progressive parkinsonism	NA	Periventricular T2/FLAIR hyperintensity Cortical atrophy	Presence of the gene region of the fusion (F) protein of measles virus in CSF Brain biopsy = neuronal degeneration, but absence of inclusion bodies	Intraventricular interferon-α and inosiplex	7 months	Her parkinsonism disappeared.

Santosh Kumar and Radhakrishnan 1998 [12]	India	25/M	NA	Measles at 24 months	4 months	Seizures Mental decline Myoclonus	Akinetic mute	NA	NA	NA	8 years	After 10 months started improving and even after 8 years she was independent

Woelfle et al. 1996 [13]	Germany	12/F	Not vaccinated	Measles at 24 months	4 weeks	Mental decline Spastic gait leading to difficulty in walking Seizures	Severe disability	Periventricular T2/FLAIR hyperintensity	NA	Antiepileptic drugs	4 years	Patient improved substantially and improvement remained sustained

Barraclough 1983 [14]	United kingdom	15/M	NA	Measles at 15 months	NA	Seizures Mental decline Myoclonus	2 years later akinetic mute	NA	Brain biopsy = gliosis, hypertrophied astrocytes, perivascular cuffing No inclusion bodies	NA	5 years	Able to walk and speak

Risk et al. 1978 [15]	USA 5 cases from the Middle East	In a Middle Eastern study, 5% of SSPE patients showed significant long-term improvement, with some surviving beyond three years. These patients displayed typical clinical signs, elevated measles antibodies, and stable or improved conditions up to 11 years post-onset.										


EEG = Electroencephalography; F = Female; FLAIR = Fluid-Attenuated Inversion Recovery; M = Male; NA = Not available; USA = United states of America. *SSPE cases those were diagnosed by Dyken’s criteria. **Long term improvement included more than 3 years of duration. Substantial Improvement/remission defined as improvement in disability score.

A study conducted by Prashanth et al. in India between 1995 and 2004 found that 19 out of 307 patients (6.2%) with SSPE experienced a relatively mild course of the disease, with survival rates exceeding three years [7]. These patients, predominantly male, had a mean onset age of 11.7 years and were followed for an average of 5.9 years. Initial symptoms varied, including seizures, myoclonus, visual disturbances, behavioral changes, and cognitive impairment. Some patients experienced stabilization for 6 months to 5 years, remissions lasting up to 9 years, and functional recovery from bed-bound to ambulatory states. In another example, Yalaz et al. [16]. documented symptomatic improvement in 50% of their study group (11 out of 22 patients) and disease stabilization in five patients following treatment with intraventricular interferons and isoprinosine. Nevertheless, extended follow-up spanning 5 to 9 years revealed that most patients who initially responded to treatment eventually exhibited neurological decline, with a significant number succumbing to the disease. Therefore, the impact of the therapy appears transient and does not seem to enhance long-term outcomes. Despite this, survival extending up to 13 years post-treatment with intraventricular interferon has been recorded [5]. Studies conducted by Anlar et al. [17,18], in Turkey, have recorded extended survival in SSPE patients undergoing treatment with interferon-alpha and isoprinosine, suggesting that proactive treatment may halt disease progression.

Gascon and co-workers also noted a subset of SSPE patients who have entered remission and outlived the typical disease course after receiving various therapies, including intraventricular interferon [19]. The exact contributors to remission remain elusive, though factors such as prompt and intensive therapy, younger age at disease onset, and possibly a less aggressive measles virus variant may play a role. The role of the immune response in SSPE’s progression cannot be understated. Variables such as the age at which SSPE manifests, the age at measles infection, and the presence of neuroimaging abnormalities seem to influence survival rates [4].

In conclusion, we present a case report of SSPE showing significant improvement, leading to a notable enhancement in the patient’s quality of life. In several studies, the effectiveness of these treatments remains uncertain due to the small sample sizes and lack of control groups. There is a pressing need for longitudinal studies to identify the subset of patients who naturally survive for extended periods. This will aid clinicians in providing better prognostic information to the patient’s relatives.
